# The Link Between Anxiety and Depression, and Balance in Young Adults

**DOI:** 10.3390/audiolres15030057

**Published:** 2025-05-12

**Authors:** Tatiana Marques, Patrícia Bernardo, Margarida Serrano

**Affiliations:** 1 Coimbra Institute for Biomedical Imaging and Translational Research, University of Coimbra, 3000-548 Coimbra, Portugal; 2 Polytechnic University of Coimbra, Rua da Misericórdia, Lagar dos Cortiços, S. Martinho do Bispo, 3045-093 Coimbra, Portugalmserrano@estesc.ipc.pt (M.S.); 3 Faculty of Medicine, University of Porto, 4200-319 Porto, Portugal; 4 H&TRC—Health & Technology Research Center, Coimbra Health School, Polytechnic University of Coimbra, Rua 5 de Outubro, 3046-854 Coimbra, Portugal

**Keywords:** anxiety, balance, depression, postural control, young adult

## Abstract

**Background/Objectives:** The ability of young adults to control their balance is generally effortless and can occur automatically with minimal cognitive involvement. However, this ability may be compromised when integration conflicts arise due to impairments in vestibular, visual, or somatosensory functions. Hence, psychomotor symptoms linked to emotional states can also influence postural control. The purpose of this study was to understand the effects of anxiety and depression on balance in young adults. **Methods:** Our study included 50 young adults (21.86 ± 2.63 years), consisting of 13 males and 37 females. Anxiety and depressive symptoms were evaluated using the Hospital Anxiety and Depression Scale (HADS), while balance was assessed through the Modified Clinical Test for the Sensory Interaction on Balance (mCTSIB). Data analysis was conducted using Pearson’s correlation coefficient test and the Kruskal–Wallis test. **Results:** Pearson’s correlation analysis indicated that young adults exhibited stable postural control. However, a positive correlation (0.259, *p* < 0.1) was observed between anxiety levels and the sway index. Additionally, positive correlations were found between anxiety and both somatosensory (0.281, *p* < 0.05) and visual (0.276, *p* < 0.1) ratios. **Conclusions:** The results suggest that higher anxiety levels are associated with reduced postural balance, with sensory inputs, particularly visual and somatosensory, playing a key role in this decreased stability.

## 1. Introduction

Anxiety and depression are distinct psychiatric disorders with an overlap of symptoms between them [1,2]. Most published studies focused on later-life anxiety and depression; however, a cross-sectional survey in the United States of America revealed that the prevalence of depression in adolescents and young adults has risen, and there is a growing number of depressed young adults who are not receiving any mental health treatment [3]. Recently, Villaume, Chen, and Adam reported that anxiety and depressive disorders affect approximately 40% and 33% of adults aged 18 to 39 years, respectively, compared with 20% and 16% of adults aged 60 years and older during the COVID-19 pandemic. After the pandemic period, levels declined for those aged 60 years and older but remained elevated for younger adults [4]. Along with these data, anxiety and early-life depression, compared with later-life depression, are characterized by less agitation, hypochondriasis, as well as less somatic symptoms, leading to an undiagnosed and underestimated prevalence of these mental conditions. Concomitantly, a strong association has been demonstrated between psychiatric comorbidity and episodic vertigo syndromes, such as vestibular migraine and Ménière’s disease in young adults [5]. The novel evidence of another separate clinical illness, persistent postural–perceptual dizziness (PPPD), also suggests a connection between vertigo symptoms and anxiety. This condition is characterized by a disruption in balance and gait that occurs long after acute vestibular symptoms and is related to deficits in postural control strategies, as well as a connection to visual dependence and emotional symptoms. The underlying mechanisms may involve maladaptive central processing, where the brain incorrectly interprets signals from the vestibular, visual, and proprioceptive systems, potentially resulting in changes in postural control. Anxiety may intensify these misinterpretations, leading to more pronounced symptoms [6,7]. However, the emotional state and related somatic symptoms, as well as depression- or anxiety-specific motor symptoms, have been underscored by several recent studies.

Functional balance plays a critical role in the performance of a wide range of human goal-directed movements, including walking, reaching, and transitioning. It refers to the integrated capacity to maintain postural stability and regulate body movements in both static positions and dynamic tasks. Effective balance control depends on the ability to keep the center of mass within the base of support, which is essential for maintaining upright posture, movement efficiency, and recovery from disturbances.

A more specific component of balance is postural control, which involves both automatic and voluntary adjustments that allow the body to sustain posture and alignment during a variety of motor tasks. This dynamic and continuous process supports coordinated movement across multiple directions, even in response to sudden changes in position. Effective postural control relies on finely tuned muscular activation and joint alignment, allowing for continuous adaptation to internal and external perturbations [8,9].

The maintenance of postural control requires the integrity of the visual system, which provides environmental and spatial orientation cues, the somatosensory system, which detects mechanical changes in muscles, tendons, and joints (proprioception), and the vestibular system, which provides information about head position and motion relative to gravity. These sensory inputs are processed and integrated by a wide range of brain areas, such as the cerebellum, brainstem, and somatosensory cortex, which work together to generate appropriate motor responses to maintain postural stability [10]. Any abnormality in any of these or an integrity conflict within the balance control system can result in the sensation of dizziness, imbalance, or vertigo [5,11]. Also, several studies reported that mismatched information between these inputs may also be related to other medical conditions, namely psychological comorbidities that impact the capacity for balance and orientation in space [5,12,13,14]. In fact, in healthy adults, movement is operated automatically under the control of the brainstem and spinal cord, which requires a small amount of cognitive resources [15]. For example, standing on a moving bus while carrying a bag is a challenging balance task that may require the involvement of higher cortical functions, such as attention and other executive functions. However, a few studies have demonstrated that this process may be affected by emotional states, due to a more complex process that leads to a higher involvement of cortical regions, and consequently to the recruitment of greater cognitive resources [5,12,13,14]. When cognitive resources are exhausted, balance instability and falls may occur [15].

Accordingly, Park et al. reported that depressive symptoms have a negative association with balance, revealing that a lower body balance score in patients with higher symptoms of depression may be explained by the underlying neurobiological and pathological mechanisms of depression, where both emotional and motor systems are affected [12]. Previously, similar findings were described by Cruz et al., who conducted a cross-sectional study, verifying that the patients with an altered dynamic balance had a higher rate of negative emotional states compared to the controls, specifically stress [5]. Moreover, Redfern, Furman, and Jacob are the authors of the first studies confirming a direct influence of anxiety in balance threat [16], followed by the recent evidence of related changes in all aspects of postural control, including standing, anticipatory, and reactive balance in anxiety disorders [17,18,19]. Indeed, individuals with anxiety-specific personality traits seem to be more conscious of their postural control, which may interfere with autonomic control processes and lead to larger amplitude postural sway when under threat [18]. Conversely, Johnson et al. suggested that a reduced allocation of attention resources to the task is the main cause of postural control deficits, resulting in less effective performance. Therefore, there is evidence that attention to movement has the potential to contribute to threat-related changes in postural control [17].

Concomitantly, these results indicate that patients with anxiety disorders are more susceptible to postural control deficits and falls, revealing a decrease in amplitude and an increase in the frequency of center of pressure (COP) displacements during quiet standing. Additionally, higher anxiety levels seem to lead to progressive decreases in sway amplitude and increases in sway frequency [20]. As previously reported, Indovina et al. highlighted a connection between personality traits and functional alterations in central vestibular pathways. These findings showed that both central vestibular and anxiety systems may be more reactive to vestibular stimuli according to personal traits [21]. Changes in connectivity patterns between subcortical vestibular and anxiety processing brain structures may elicit stronger neuronal reactions to vestibular functions, threatening stimuli in anxiety-related personality traits; however, methodological differences in the assessment of the emotional state and balance can be limited by the use of self-reported emotional state and different balance assessment approaches, with limited evidence into this relationship.

On the other hand, the different involvement of emotional processes, i.e., anxiety, depression, stress, panic disorder, and others, determines the necessity of ongoing research to better understand the etiology beyond this relationship, which could help to develop vestibular rehabilitation that focuses on sensory re-integration processes, including processing sensory information that is significantly influenced by threat. Therefore, it is crucial to understand how emotional factors can influence balance control, as these changes have the potential to mask or modify underlying balance deficits.

Given the trends in the prevalence of anxiety and depression in young adults in the last few years, and consequently the growing number of young people with untreated depression, we expected that anxiety and depression would influence balance in young adults without any clear physiological dysfunction. Therefore, the present study aimed to examine the postural control in this population.

## 2. Materials and Methods

Ethical approval was obtained in September 2023 from the Ethics Committee of Polytechnic Institute of Coimbra (approval number 108_CEIPC/2023), and all participants provided written and verbal informed consent. This study was conducted in the Audiology Laboratory of the Coimbra Health School.

### 2.1. Participants

A total of 50 young adults participated in the study, with an average age of 21.86 ± 2.6 years, of which 13 were males and 37 were females. Briefly, exclusion criteria for the study were a history of neuropsychiatric disease besides anxiety or depression, any other vestibular impairment, injury (including previous lower limb surgery) or medication that possibly affects balance, and outer and middle ear pathologies.

### 2.2. Procedure

Participants completed a self-administered questionnaire, which included information on their demographic and clinical history, as well as their personal habits (e.g., physical activity; smoker or not). After the initial assessment, a self-administered questionnaire on symptoms related to vestibular disorders was given. It includes items with yes/no responses, as well as a set of written questions designed to describe sensations of dizziness, imbalance, or vertigo and their duration, if applicable.

The HADS was developed by Zigmond and Snaith [22], and there was a Portuguese version translated and validated by Pais-Ribeiro et al. that was used in this study [23]. The scale is a self-administered instrument designed to provide clinicians with a reliable, valid, and user-friendly tool for identifying and quantifying symptoms of anxiety and depression. Initially, it was intended to identify hospital patients who may require further psychiatric evaluation and intervention; however, nowadays, it is broadly used in clinical practice as a tool to screen for anxiety and depression, rather than diagnose specific psychiatric disorders [24]. Therefore, the HADS was used to evaluate the severity of anxiety and depression, comprising 14 questions, scored from 0 to 3. Seven questions pertain to anxiety (maximum score of 21), and seven questions pertain to depression (maximum score of 21), with scores for each subscale between 0 and 7 indicating the absence of symptoms, scores between 8 and 10 indicating mild symptoms, scores of 11 to 14 indicating moderate symptoms, and scores of 15 to 21 indicating severe symptoms.

#### 2.2.1. Evaluation of Postural Stability

Postural control was assessed using the mCTSIB. The evaluation was conducted on the computerized posturography platform NeuroCom, model Basic Balance Master System Version 8.2.0. Computerized posturography is one of the most frequently used methods in both clinical practice and research for assessing postural control. It allows for the evaluation of sensory contributions under manipulated sensory conditions, as seen in protocols such as the Sensory Organization Test (SOT) and the Modified Clinical Test of Sensory Interaction on Balance (mCTSIB) [8,9].

One of the primary advantages of computerized posturography is its ability to produce objective, quantifiable measurements of balance performance. The system uses a force platform equipped with four load cells that collect force data at a high frequency—typically 100 Hz (100 samples per second). These data are processed to calculate the center of pressure (COP) and, using the patient’s height, estimate the vertical component of the center of gravity (COG). The movement and positioning of the COG are monitored continuously, allowing for the precise analysis of postural sway and stability under various conditions [25,26].

The mCTSIB replicates the Sensory Organization Test by using a compliant foam pad instead of a sway-referenced forceplate. Timed measurements are taken using a stopwatch to provide scores. The test aims to identify abnormalities in the three sensory systems that contribute to postural control: somatosensory, visual, and vestibular.

Participants completed the mCTSIB assessment by standing quietly on the forceplate, in four different sensory conditions: (1) firm surface, eyes open (F/EO); this serves as the baseline for comparison with the other three conditions. (2) Firm surface, eyes closed (F/EC); to maintain stability, reliance is primarily on somatosensory inputs and, secondly, on vestibular inputs. (3) Foam surface, eyes open (FO/EO); primarily relies on visual inputs and secondarily on vestibular inputs. (4) Foam surface, eyes closed (FO/EC); vestibular information remains available and accurate. To maintain stability, the individual will mainly depend on vestibular inputs [8,9]. Participants were instructed to stand in each testing condition for 10 s, with three trials conducted under each condition. The test was administered without shoes.

The mCTSIB variable of interest was the average sway velocity in each of the conditions and the sway index. The sway index represents the average of the mean sway velocity scores for all conditions. Further, sensory ratios were calculated for each participant. For the somatosensory ratio, the sway velocity of F/EC is divided by the sway velocity of F/EO; for visual, the sway velocity of FO/EO is divided by the sway velocity of F/EO; and for the vestibular ratio, the sway velocity of FO/EC is divided by the sway velocity of F/EO. Sensory ratios indicate the ability to reweigh sensory input and place a stronger reliance on the predominant system for each ratio [8].

#### 2.2.2. Statistical Analysis

Descriptive statistics were performed to measure central tendency, including mean, median, maximum, minimum, and standard deviation (SD) for continuous data, or N and percentage (%) for discrete variables. Correlations were assessed among variables using Pearson’s correlation coefficient test at a significance level of 0.05. *p*-values between 0.05 and 0.1 were considered marginally significant. The Kruskal–Wallis test was used to compare the results of postural control evaluation across various levels of anxiety and depression.

## 3. Results

### 3.1. Participants’ Demographic and Clinical Characteristics

The sample included fifty participants whose demographic and clinical characteristics are listed in Table 1. The mean age of the participants was 21.86 (SD = 2.63) years.

Of the fifty young adults who participated in this study, we noticed a higher participation of the female gender (74%) as well as that only ten participants were employed. A lifestyle variable analyzed was physical activity, and when asked whether they had participated in some form of physical activity, 38% reported having participated in regular physical activity. Of the young adults included, 13 (26%) were smokers. Furthermore, none of the participants had a self-reported history of vestibular disease.

**Figure 1:**
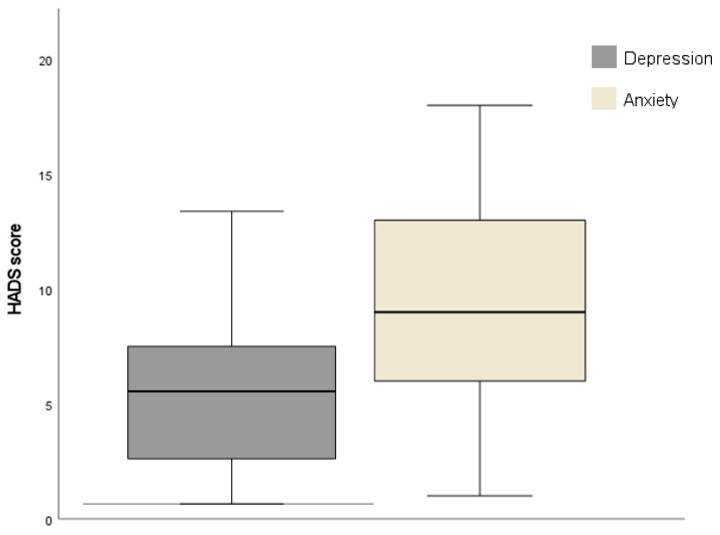
Distribution of HADS scores on anxiety and depression subscales.

The mean HADS—anxiety scores were above the cut-off of the 8+ criterion for identifying cases of anxiety, indicating an anxiety prevalence of 62%, of whom 48.4% showed moderate symptoms. In detail, the distribution of the HADS—anxiety score was 9 (SD 4.63; range 1–18). On the other hand, on the depression subscale, a mean of 5.26 (SD 3.44; range 0–13) was obtained, as shown in Figure 1. Concerning depression subscale scores, participants exhibit fewer and less severe depressive symptoms, as can be observed in Table 2. Nonetheless, the results of Pearson’s correlation showed a moderate correlation with an increase in the HADS—anxiety scores and the HADS—depression scores (r = 0.608, *p* < 0.001).

Correlation analyses were performed to examine the relationship between the HADS scores and demographic and clinical characteristics. A significant correlation was found exclusively between gender and anxiety (r = 0.346, *p* = 0.014), with females being more anxious than males.

### 3.2. Postural Stability

The means for sway velocity for all mCTSIB conditions are reported in Table 3. As expected, the sway velocity mean increased significantly (*p* < 0.001) as a result of increasing task difficulty on postural control, showing that participants had more difficulties in the eyes-closed standing on foam condition. However, the Kruskal–Wallis analysis between anxiety levels and mCTSIB conditions revealed that sway postural control is significantly different between anxiety participants when visual and somatosensory cues are unavailable (FO/EO condition: mild vs. severe anxiety, *p* = 0.034, and moderate vs. severe anxiety, *p* = 0.011; FO/EC condition: mild vs. severe anxiety, *p* = 0.027, and moderate vs. severe anxiety, *p* = 0.035).

The correlation analysis showed no significant correlation between the HADS–anxiety scores and the mCTSIB conditions (see Table 4), as well as between the HADS—depression scores, the mCTSIB conditions, and the sway index. However, weak positive correlations between anxiety symptoms and the somatosensorial ratio (r = 0.28, *p* = 0.048), and anxiety symptoms and the visual ratio (r = 0.28, *p* = 0.05) were found. Participants exhibited a marginally significant correlation between anxiety and the sway index (r = 0.26, *p* = 0.069). For the depression subscale, we did not find significant correlations.

**Figure 2:**
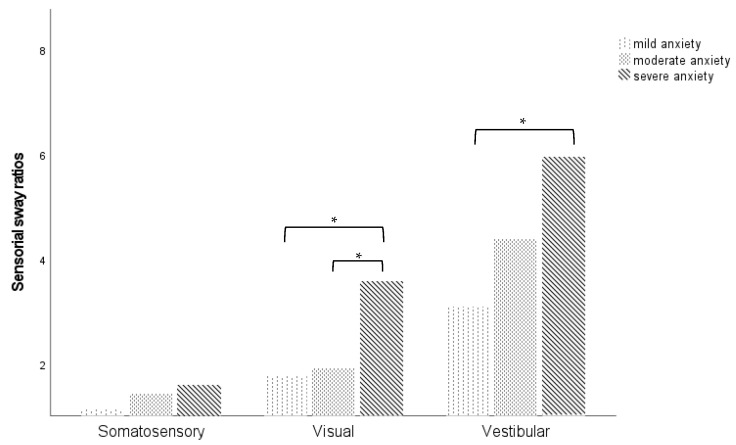
Average sway velocities by mCTSIB-specific somatosensory, visual, and vestibular ratios according to anxiety levels. * *p* < 0.05.

Moreover, individuals who showed severe symptoms of anxiety reported a higher postural sway velocity for all sensory ratios, especially for visual (mild vs. severe anxiety, *p* = 0.042; moderate vs. severe anxiety, *p* = 0.019) and vestibular ratios (mild vs. moderate anxiety, *p* = 0.071; mild vs. severe anxiety, *p* = 0.047), as shown in Figure 2.

Among depression scores, the mean values of sway velocity for sensory ratios were similar between participants. Analyzing the HDAS—depression scores and mCTSIB sensory ratios, no correlation was found. Table 4 shows the correlation between variables.

## 4. Discussion

The present experiment explored whether one’s emotional state, i.e., anxiety and depression, can influence static balance among young adults. First, it is important to notice that high levels of anxiety were identified in this population, with a prevalence of 62%. Villaume et al. had previously obtained similar results, concluding that young adults have higher levels of depression and anxiety than older adults [4]. Hence, it is extremely important to evaluate the emotional state across the lifespan, and specifically in young adults, to be aware of other comorbidities that may be the cause or caused by neuropsychiatric disorders.

Second, we expected that higher levels of anxiety or depressive symptoms would be associated with increased postural sway. Generally, young adults from our sample had reliable postural control; nevertheless, we found a positive correlation between anxiety levels and the sway index. These findings suggest that an increase in the HADS—anxiety score is associated with a higher sway index score, indicating a greater degree of instability and an increased risk of fall.

Moreover, our findings from sensorial sway ratios revealed a positive association of anxiety and somatosensorial and visual ratios. Specifically, the higher the anxiety score on the HADS, the greater the use of somatosensory or visual references, if visual or somatosensory cues, respectively, are removed. In comparison between anxiety levels, in unstable conditions of the mCTSIB, it was observed that higher scores of anxiety lead to more difficulties in postural control, especially when, besides somatosensorial cues, visual cues are inaccurate. Therefore, anxiety seems to increase reliance on somatosensory and vision systems for balance due to a decreased ability to use vestibular feedback for balance. These results support the findings of Goto et al., who evaluated the effect of anxiety on the postural stability of 54 patients with dizziness and found that higher levels of anxiety are associated with greater postural sway. Furthermore, it was identified that anxiety affects the interactions of visual, on vestibular, and somatosensory inputs in the maintenance of postural control [27]. However, these authors studied postural control in patients complaining of dizziness, with a significant effect of the vestibular perturbation.

Furthermore, the diminished performance of postural control may be explained by changes in postural threat, i.e., inaccurate visual or somatosensorial cues, leading to more anxiety and consequently affecting postural control measures. Therefore, we hypothesized that postural threat emerges as a consequence of higher anxiety related to the possibility of instability, such as what may occur when there is a fear of falling. This interpretation is in line with the study of Hauck, Carpenter, and Frank, who suggested that increased levels of threat and consequently of task difficulty led to decreased stability, while anxiety levels increased [19]. Moreover, increased motor task difficulty may exert greater cognitive resources, as has been demonstrated previously by Khaya et al., who showed that a challenging postural demand is associated with cognitive overload in healthy young adults. Therefore, if postural threat is mediated by anxiety, and knowing that anxious thoughts affect cognitive resources due to overloading [11,28], leading to competitive processes, these results may suggest that postural control is affected by anxiety due to overloaded cognitive resources in young adults. These findings are of clinical importance, namely due to the high prevalence of psychiatric comorbidities in the young population. Hence, Stins, Roerdink, and Peek demonstrated that affective interventions could contribute to ameliorating postural control, which could be a potential tool to introduce in vestibular rehabilitation [18]. Conversely, when the severity of the symptoms of anxiety increase, both vision and vestibular functions provide critical information for postural control. The reason for the lack of a correlation in the somatosensorial sway ratio and greater dependence on vestibular cues for postural control may be related to the stiffness of their stance under threat that is worse in proportion with the severity of the anxiety symptoms, as well as the personality traits associated with anxiety, specifically neuroticism and introversion, which may be important factors for anxiety-mediated vestibular conditions. Accordingly, Hacohen-Brown et al. showed that patients with more severe symptoms are also more likely to experience hypersensitivity to vestibular stimuli and show greater dependence on vestibular cues for postural control, possibly due to poor somatosensorial control or altered visual–vestibular integration [29].

Surprisingly, for depression, no significant interaction was found, even considering that the HADS subscales’ scores are significantly correlated. Considering that in our study, young adults had fewer and less severe symptoms of depression, these results may be different if the depression symptoms are worse. In fact, Park et al. have shown that a higher clinical risk for depression has a negative association with balance scores due to dopamine pathway dysregulation, which leads to motor symptoms specific to depression [12]. Other studies suggested that, specifically, dynamic balance is affected by emotional states; however, other comorbidities and lifestyle factors are also implicated as a potential cause of balance dysfunction [5]. In fact, in this study, we compared HADS scores and demographic and clinical characteristics, namely physical activity, and no association was found. However, Cruz et al. did not use an objective instrument to assess depression, which reduces the reliability and replication of their results.

While this study provides important preliminary evidence, it has some limitations. In fact, even considering that we have observed some significant differences or associations, these need to be interpreted with caution given the relatively small sample size and small-to-moderate effect sizes. Furthermore, our study design included an unequal ratio of both females and males, and there are well-known differences between gender in spatial anxiety, with males outperforming females [30], as well as a higher prevalence of anxiety in females, which could have influenced our results. Furthermore, our findings highlighted the necessity of establishing an intervention program targeted toward young adults. Despite frequently being underdiagnosed with anxiety or depression, these individuals show a clear need for support and an increased awareness of these conditions, which have worsened and have been overlooked following the COVID-19 pandemic period. Intervention programs, such as vestibular rehabilitation or mindfulness practices, including meditation, yoga, and deep breathing exercises, have been shown to help manage anxiety and enhance balance. These interventions have the potential to significantly improve the quality of life for young individuals affected by anxiety and depression, as highlighted in recent studies [31,32].

Future work should address the reliability of these results in terms of gender, as well as in psychiatric populations, specifically in patients with anxiety-specific personality traits. Finally, a more precise assessment of anxiety and depression may be necessary, suggesting combining scales with clinical interviews and behavioral observations.

## 5. Conclusions

Increased postural sway and consequently a greater degree of instability and an increased risk of fall were associated with higher levels of anxiety, which reflects a higher dependence on the somatosensory and vision systems for maintaining postural control. Otherwise, depression symptoms do not seem to influence postural control. This implication is in direct contradiction with extensive research that showed evidence that depression is detrimental to balance. We suggest that potential sources of anxiety are associated with increased levels of perceived threat due to a decreased ability to use vestibular feedback for balance.

Future work in this field will provide insights into this association and the reliability of these results.

## Figures and Tables

**Table 1:** Participants’ demographic characteristics.

		N (%) or Mean (SD)	Min	Max
Age		21.86 (2.63)	18	32
Gender	Male	13 (26%)	-	-
	Female	37 (74%)	-	-
Employment status	Student	40 (80%)	-	-
	Employed	10 (20%)	-	-
Physical activity	No	31 (62%)	-	-
	Yes	19 (38%)	-	-
Smoke	No	37 (74%)	-	-
	Yes	13 (26%)	-	-

Abbreviations: N, number; %, percentage; SD, standard deviation.

**Table 2:** Distribution of anxiety and depression levels according to HADS scores.

		N (%) or Mean (SD)
Anxiety levels (N = 31)	Mild	10 (32.3%)
	Moderate	15 (48.4%)
	Severe	6 (19.4%)
Depression levels (N = 12)	Mild	7 (58.3%)
	Moderate	5 (41.7%)
	Severe	-

Abbreviations: N, number; %, percentage; SD, standard deviation.

**Table 3:** Mean sway velocity for the mCTSIB.

	Mean (SD)	Min	Max	DP
F/EO	0.17	0.10	0.40	0.08
F/EC	0.19	0.10	0.40	0.07
FO/EO	0.33	0.20	0.80	0.17
FO/EC	0.61	0.20	1.20	0.23
Sway index	0.33	0.20	0.80	0.15

Abbreviations: F/EO: firm, eyes open; F/EC: firm, eyes closed; FO/EO: foam, eyes open; FO/EC: foam, eyes closed.

**Table 4:** Pearson’s correlation coefficients among anxiety, depression, and mCTSIB.

	Anxiety	Depression
F/EO	−0.017	−0.053
F/EC	0.210	−0.044
FO/EO	0.199	0.43
FO/EC	0.160	0.016
Sway index	0.259 *	−0.026
Somatosensory	0.281	0.137
Visual	0.276 *	0.083
Vestibular	0.186	0.064

Abbreviations: F/EO: firm, eyes open; F/EC: firm, eyes closed; FO/EO: foam, eyes open; FO/EC: foam, eyes closed. * *p* < 0.05.

## Data Availability

The raw data supporting the conclusions of this article will be made available by the authors on request.

## Abbreviations

The following abbreviations are used in this manuscript:

F/EO	Firm/eyes open
F/EC	Firm/eyes closed
FO/EO	Foam/eyes open
FO/EC	Foam/eyes closed
HADS	Hospital Anxiety and Depression Scale
mCTSIB	Modified Clinical Test for the Sensory Interaction on Balance
SD	Standard deviation
